# A universal metasurface transfer technique for heterogeneous integration

**DOI:** 10.1515/nanoph-2022-0627

**Published:** 2023-01-11

**Authors:** Xu Zhang, Haogang Cai, Soroosh Daqiqeh Rezaei, Daniel Rosenmann, Daniel Lopez

**Affiliations:** Department of Electrical and Computer Engineering, Carnegie Mellon University, Pittsburgh, PA 15213, USA; Department Tech4Health Institute and Department of Radiology, NYU Langone Health, New York, NY, 10016, USA; Department of Electrical Engineering & Materials Research Institute, The Pennsylvania State University, University Park, PA, 16802, USA; Center for Nanoscale Materials, Argonne National Laboratory, Lemont, IL, 60439, USA

**Keywords:** heterogeneous integration, metasurface, nanofabrication, transfer

## Abstract

Metasurfaces offer a versatile platform for engineering the wavefront of light using nanostructures with subwavelength dimensions and hold great promise for dramatically miniaturizing conventional optical elements due to their small footprint and broad functionality. However, metasurfaces so far have been mainly demonstrated on bulky and planar substrates that are often orders of magnitude thicker than the metasurface itself. Conventional substrates not only nullify the reduced footprint advantage of metasurfaces, but also limit their application scenarios. The bulk substrate also determines the metasurface dielectric environment, with potentially undesired optical effects that undermine the optical performance. Here we develop a universal polymer-assisted transfer technique to tackle this challenge by decoupling the substrate employed on the fabrication of metasurfaces from that used for the target application. As an example, Huygens’ metasurfaces with 120 nm thickness in the visible range (532 nm) are demonstrated to be transferred onto a 100 nm thick freestanding SiN_*x*_ membrane while maintaining excellent structural integrity and optical performance of diffraction-limited focusing. This transfer method not only enables the thinnest dielectric metalens to the best of our knowledge, but also opens up new opportunities in integrating cascaded and multilayer metasurfaces, as well as the heterogeneous integration with nonconventional substrates and various electronic/photonic devices.

## 1 Introduction

The emergence of metasurfaces serves as an immense driver for innovative technologies and applications including dispersion engineering [1], polarization manipulation [2], metasurface-assisted augmented and virtual reality (AR/VR) [3, 4], compact microscopy and spectroscopy [5, 6], computing [7, 8], information encryption and display [9, 10], structural color generation [11–14], etc. A significant obstacle hampering this progress, however, is challenges in fabrication and integration. Well-established nanofabrication techniques often require standard bulky substrates that are often several orders of magnitude thicker than the fabricated nanostructures and primarily playing a mechanical role to maintain the metasurface integrity. However, the bulky substrates also impose optical effects, which break the symmetry of the surrounding dielectric environment, and reduce the refractive index contrast between the meta-atoms and surrounding medium. The substrate effects could result in the decline of meta-atom resonance Q factors of and metasurface optical function efficiencies [15]. In addition, this inherent problem nullifies the reduced footprint advantage provided by metasurfaces, which is replacing bulky optical assemblies.

More importantly, with the growth of metasurface-driven devices, there is a pressing need for heterogeneous integration of metasurfaces on top of non-conventional substrates or optical/electronic elements that are not possible using current fabrication processes. For example, in order to minimize the undesired substrate effects, an optically “free-floating” metasurface can be achieved by placing the metasurface on a thin film with low refractive index [15]. The requirement for metasurface optical integration is beyond simple doublets on the two sides of bulky substrates [16, 17] or macroscopic assembly of multiple meta-optical elements [18, 19]. Tightly-spaced cascade metasurfaces have been used to enable direction-dependent optical functions, such as asymmetric transmission [20]. Multilayer integration has been explored to enrich the interactions within and between layers for increased functionalities [21]. Further integration with electronics and 2D materials can also enable new active metasurfaces [22, 23]. The integration with membranes [24], thin films and electronics is possible but remains challenging, considering fabrication compatibility and aggressive etching and planarization processes.

Non-conventional substrates are further extended from uniform and rigid planar surfaces to non-planar or flexible surfaces. For example, meta-fibers with metasurfaces on optical fiber tips promise enormous potential in areas like communications, imaging and sensing for various applications from quantum technology to health care [25–27]. Nanofabrication on a fiber tip is generally not compatible with the most popular electron-beam lithography, and relies on other *in-situ* techniques such as focused ion beam (FIB) [26] and 3D printing [27, 28]. Metasurface integration on flexible substrates enables not only conformal or wearable photonic devices [29, 30], but also dynamic metasurfaces with mechanical [31] or electrical [32] actuation. However, the flexible substrates have been generally limited to certain polymer materials including polydimethylsiloxane (PDMS), photoresist such as SU-8, through a “pick-up” process relying on suspended structures or sacrificial layers. Besides plasmonic metals [31], flexible dielectric metasurfaces have been limited to Si [30, 32], chalcogenide (ChG) glass [29, 33] materials for infrared (IR) light. Overall, the target substrates used for specific applications are often not the ideal substrates for metasurface fabrication. In order to fully unleash the potential of metasurfaces, it is critical to develop a universal and scalable transfer methodology to decouple the target application substrates from optimum fabrication substrates. Such a transfer technique should also maintain nanoscale structural integrity during the transfer process, so that the metasurfaces will realize the desired optical functions after being transferred onto the target substrates.

In this work, we demonstrate a polymer-assisted transfer technique that allows decoupling the target substrate from the fabrication substrate and transferring metasurfaces onto arbitrary substrates with high fidelity. Based on the releasing of a water-soluble sacrificial layer, metasurfaces can be embedded in the protective polymer thin films, and further transferred onto a variety of target substrates with arbitrary configurations as aforementioned, e.g., freestanding membranes, cascaded and multilayer surfaces, optical fiber tips, etc. The polymer-based films can be easily removed, leaving metasurfaces intact on target substrates. Without the need of aggressive etching or planarization, this process is compatible with established CMOS processes while offering unprecedented design flexibility. Compared with the transfer printing of plasmonic nanogap devices [34], our simple and universal technique further extends applicable nanostructures from metallic to dielectric materials for metasurfaces. As an example, the proposed technique is demonstrated by transferring metalenses for the visible light to ultrathin freestanding membranes. Metalenses are the most popular and representative meta-optical elements [35, 36]. Departing from existing flexible IR metasurfaces based on Si or ChG, we use TiO_2_ which exhibits a high refractive index with negligible loss in the visible range. We employ Huygens’ principle to design nanodisks that offer adequate phase shifts while maintaining high transmittance. Huygens metasurfaces provide ultrathin footprint circumventing the requirement for high aspect ratio structures and enable full compatibility with standard industrial fabrication techniques. Due to their particular technological interests, here we used Huygens metasurfaces as a prototypical case to demonstrate the effectiveness of the polymer-assisted transfer technique. Nevertheless, it should be pointed out that the established transfer technique is not limited to TiO_2_ Huygens metalenses on thin membranes, and can be utilized in the heterogeneous integration of metasurfaces in different application scenarios. The transfer method presented here is also scalable and can be extended for wafer-scale mass-production of metasurfaces.

## 2 Results and discussion

**Figure 1:**
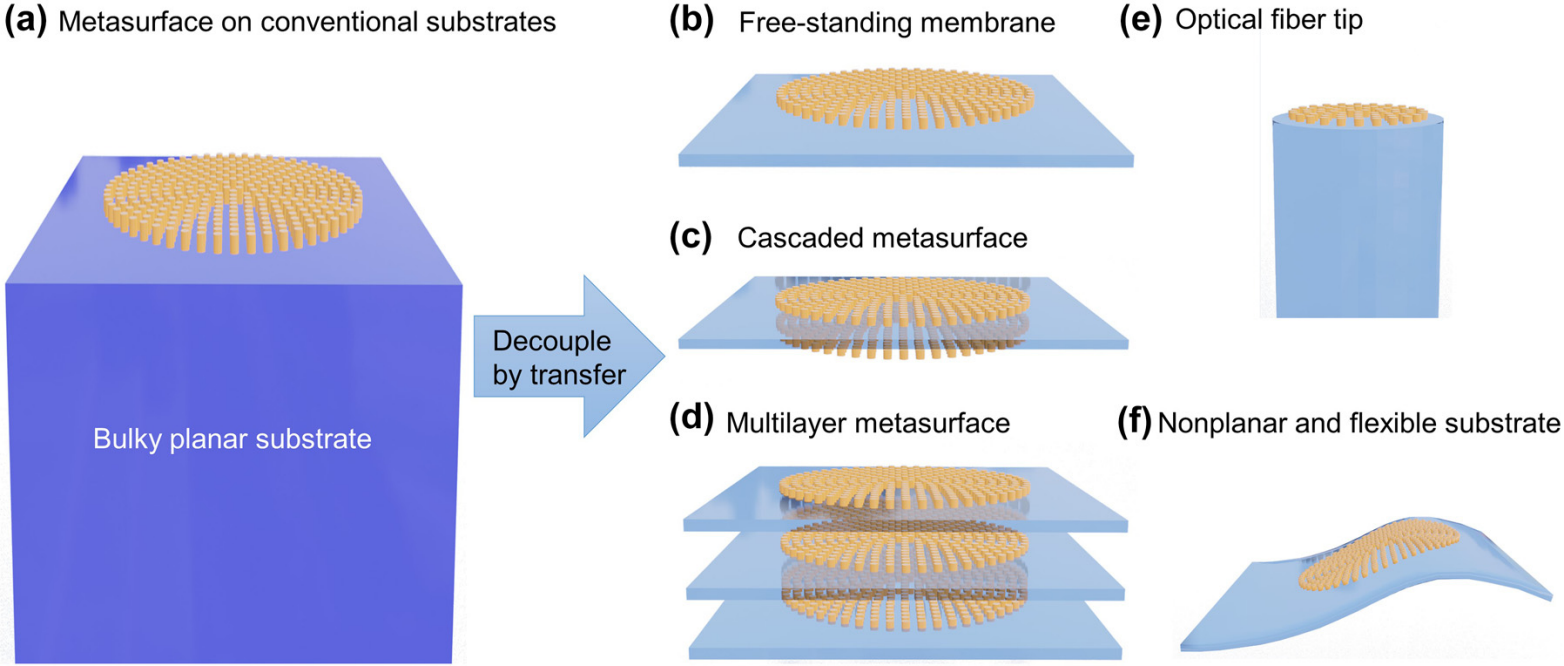
Schematic of the transfer method that decouples the fabrication substrate from the target substrate. (a) *in-situ* fabrication on conventional substrates. The transfer method allows heterogeneous integrations: (b) metasurface on freestanding membrane, (c) cascaded and (b) multilayer metasurfaces, metasurface on (e) optical fiber tip, (f) nonplanar and flexible surfaces.

Metasurfaces are predominantly fabricated on standard substrates (e.g., Si, glass), exceeding the thickness of the metasurface by several orders of magnitude. These substrates have bulky footprint and undesired optical effects, which severely hinder the adaptation of metasurfaces as functional optical elements. Figure 1 schematically illustrates that a metasurface can be decoupled from the bulky substrate by a transferring process (Figure 1a). Not only the bulky substrate is removed, but also the metasurface can be transferred to a plethora of target surfaces, which are not compatible with conventional fabrication processes, including freestanding membranes (Figure 1b), cascaded (Figure 1c), and multilayer surfaces (Figure 1d), nonplanar (Figure 1e) and flexible surfaces (Figure 1f). Such an approach could decouple metasurfaces from limited substrate types, circumvent the need for *in-situ* fabrication, and enable heterogeneous integrations that were not possible before. In the following, we demonstrate how to design and fabricate metalens on a standard bulk substrate, and transfer it to a target substrate as a proof-of-principle. Further characterization is performed to validate that the meta-atom nanostructures and their arrangement remains unchanged and that the meta-optical elements are fully functional after the transfer process.

**Figure 2:**
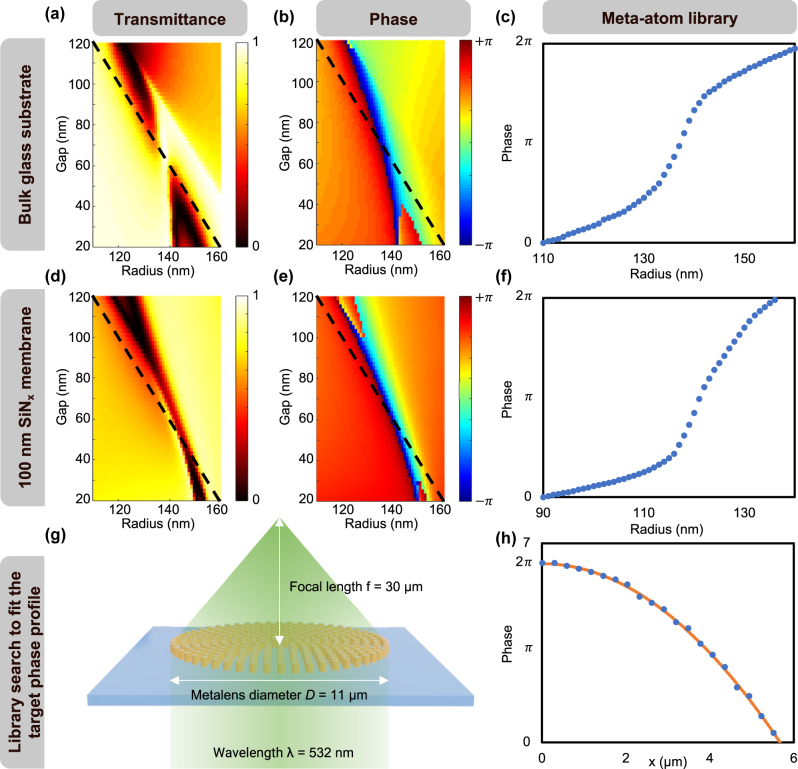
Metalens simulation and design. (a) Transmittance and (b) phase shift maps of the periodic TiO_2_ nanodisk arrays as a function of geometrical parameters radius and gap on a glass substrate. (c) Phase shift as a function of nanodisk radius for a given pitch of 340 nm on a glass substrate. (d) Transmittance and (e) phase shift maps of the periodic TiO_2_ nanodisk arrays as a function of geometrical parameters radius and gap on a 100-nm-thick SiN_*x*_ membrane. (f) Phase shift as a function of nanodisk radius for a given pitch of 290 nm on a 100-nm-thick SiN_*x*_ membrane. (g) Schematic diagram of metalens on membrane and its design parameters. The designed NA is 0.18. (h) Meta-atoms are selected from the library to fit the target phase profile of a convex lens.

As an example and proof-of-principle, we choose a free-standing SiN_*x*_ membrane as the target substrate. SiN_*x*_ membranes with various thickness and dimensions are widely used as transmission electron microscope (TEM) grids. The transfer process using readily available TEM grids avoids the need of toxic etchant or long etching process. We begin the metalens design by simulating the transmittance and phase shift from individual TiO_2_ nanodisk arrays of various gaps *g*, and radius *r* on glass (standard bulk substrate) and SiN_*x*_ (target membrane substrate) at 532 nm wavelength. The height of nanodisks *h*, is fixed at 120 nm. The transmittance and phase shift maps as a function of nanodisks radius and gap are simulated in Figure 2a–b for glass substrate. As can be observed for a fixed pitch p = 2*r* + *g* of 340 nm, changing the radii from *r* = 110 nm to *r* = 160 nm yields a full transmittance-phase coverage of 2π while maintaining near-unity transmission. High transmission efficiency along with the 2π phase shift indicates the fulfillment of Huygens’ conditions. Huygens’ metasurfaces possess high transmission by employing electrical and magnetic dipoles that are in phase with equal amplitude. Such resonators cancel the back-scattering in the incidence direction, allowing only forward propagation, as required by the Huygens principle. Figure 2c further highlights the full phase coverage by varying the radius of the nanodisks at a fixed pitch of p = 290 nm for a range of radii from 100 nm to 150 nm. This nanodisk set serves as a library to design metalens on a glass substrate.

This library, however, cannot be used for metalens design on a different substrate, which determines the dielectric environment. Meta-atom resonances and the whole library are dependent on the dielectric environment and should be developed taking into account the specific target substrate. For a 100 nm-thick SiN_*x*_ membrane, the transmittance and phase map simulations are totally different, as depicted in Figure 2d–f. Despite the full phase control, the transmittance drops for a narrow window of radius range, indicating the Huygens condition is not fully satisfied. This is probably attributed to the relatively high refractive index of SiN_*x*_. By comparing Figure 2c–d, we can see that for metalens design it is crucial to take the target substrate into consideration. As conventional bulk glass and thin membrane result in a totally different meta-atom library and geometric (disc radius) range. Figure 2g schematically illustrates metalens on membrane and its design parameters. The designed numerical aperture (NA) is 0.18. Figure 2h plots metalens phase profile fitted by TiO_2_ nanodisks from Figure 2f to a conventional convex lens profile. Based on the target phase profile, meta-atoms with different radii were selected from this library.

**Figure 3:**
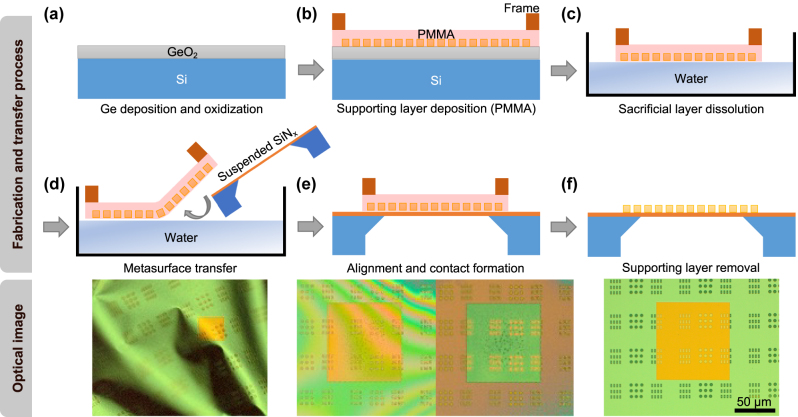
Fabrication and transfer process flow. (a) Si substrate with a 400-nm GeO_2_ is used for TiO_2_ metasurface fabrication. (b) PMMA film is spincoated and baked to encapsulate the fabricated metalens, followed by mounting kapton tapes onto the edges of sample. (c) The sample is immersed in DI water dissolving GeO_2_ and releasing the encapsulated metalens. (d) Top: the floating encapsulated metalens is transferred onto the target substrate. Bottom: optical image of encapsulated metalens being transferred onto a target substrate, a suspended SiN_*x*_ membrane with 100 nm thickness on a Si frame. (e) Top: the metalens is aligned on top of the target substrate and baked to improve adhesion. Bottom: optical images of encapsulated metalens forming conformal contact with the target substrate. (f) Top: the PMMA is dissolved by acetone completing the transfer process. Bottom: optical image of an example of the post-transfer metasurface arrays on a SiN_*x*_ membrane.

Next, the metalens arrays are fabricated on a Si substrate, and the transfer method is demonstrated as depicted in Figure 3. The fabrication begins with a 400-nm thick Ge film deposited onto a Si substrate by electron beam evaporation. The Ge film was subsequently oxidized into GeO_2_ to be used as the sacrificial layer (Figure 3a). The GeO_2_ serves as a water-soluble sacrificial layer and avoids the use of harsh chemical etchants that may damage the metasurfaces during the transfer process. TiO_2_ metasurfaces were fabricated on top, as explained in the Methods section. Then, a polymer of poly(methyl methacrylate) (PMMA) layer was spin-coated and baked on metasurfaces completely encapsulating them as a robust supporting layer for transfer. PMMA is selected here due to its excellent flexibility, mechanical robustness and the ability to form a uniform and adhesive contact with metasurface nanostructures. Kapton tapes were then mounted on the edges of the sample, serving as a frame to further minimize deformation and wrinkles of the flexible PMMA film and to facilitate manipulation during the transfer process (Figure 3b). In order to transfer the metalens, samples were immersed in a deionized (DI) water bath at 70 °C for about 10 h resulting in the dissolution of the GeO_2_ film. As the GeO_2_ layer is dissolved, the metasurface/PMMA assembly layer delaminates from the Si substrate, eventually floating on top of the wafer surface (Figure 3c). The use of water, instead of strong chemicals, to remove the sacrificial layer makes the fabrication substrate recyclable, rendering the process cost-effective. Subsequently, the encapsulated metasurface/PMMA assembly layer was transferred onto our target substrate, a suspended SiN_*x*_ membrane with 100 nm thickness on a Si frame (Figure 3d). The optical image shows an example of a transfered PMMA film with wrinkles, which can be avoided by using the Kapton frame. Figure 3e shows the process of the metasurface/PMMA layer forming conformal contact with the target substrate. During this step, the orientation and position of the metasurface arrays can be manipulated under a microscope and the kapton frame provides handles to ease the process of alignment. After the transfer, the metasurface/PMMA layer was baked at 80 °C for about 30 min to improve its adhesion with the target substrate. Finally, the PMMA layer was dissolved in acetone, thus completing the transfer process (Figure 3f). The optical image shows one example of the post-transferred metasurface arrays on a SiN_*x*_ membrane.

**Figure 4:**
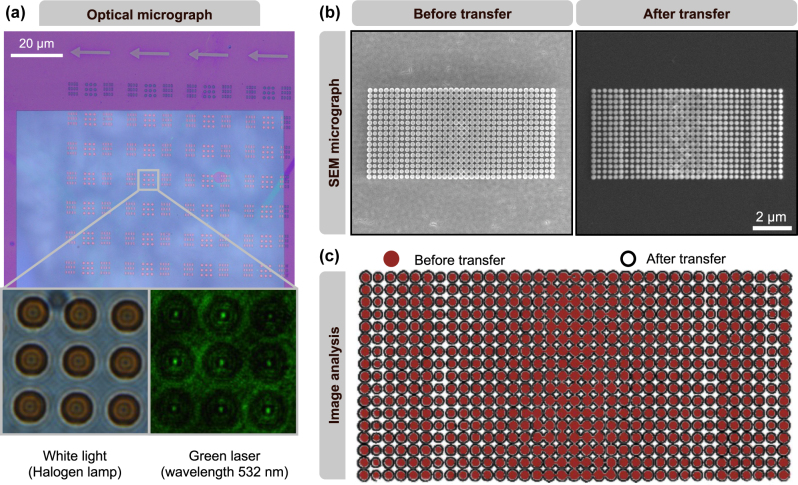
Comparison of metalens before and after transfer. (a) Optical images of the metalens arrays transferred onto a SiN_*x*_ membrane. Bottom left: imaged using white light. Bottom right: imaged using monochromatic light at 532 nm wavelength. (b) Scanning electron microscopic (SEM) image of the metalens before and after transfer onto a SiN_*x*_ membrane. (c) Comparison of the meta-atom positions before and after transfer.

Post-transferred Huygens’ metalens arrays were further characterized by optical and scanning electron microscopy (SEM) as shown in Figure 4. Optical images of the transferred arrays onto the target substrate of SiN_*x*_ membrane along with a magnified array imaged with both white light (bottom left) and monochromatic light at 532 nm wavelength (bottom right) are depicted in Figure 4a. In order to confirm the high fidelity of the transfer technique, we carried out scanning electron microscopy (SEM) characterization of the TiO_2_ metasurfaces before and after the transfer (Figure 4b). By performing image analysis on SEM images, one can determine the position of individual meta-atoms (nanodisks) and overlap them before and after the transfer as illustrated in Figure 4c. As can be observed, the position of nanodisk arrays matches very well before and after transfer, highlighting robustness and high fidelity of the proposed transfer technique.

**Figure 5:**
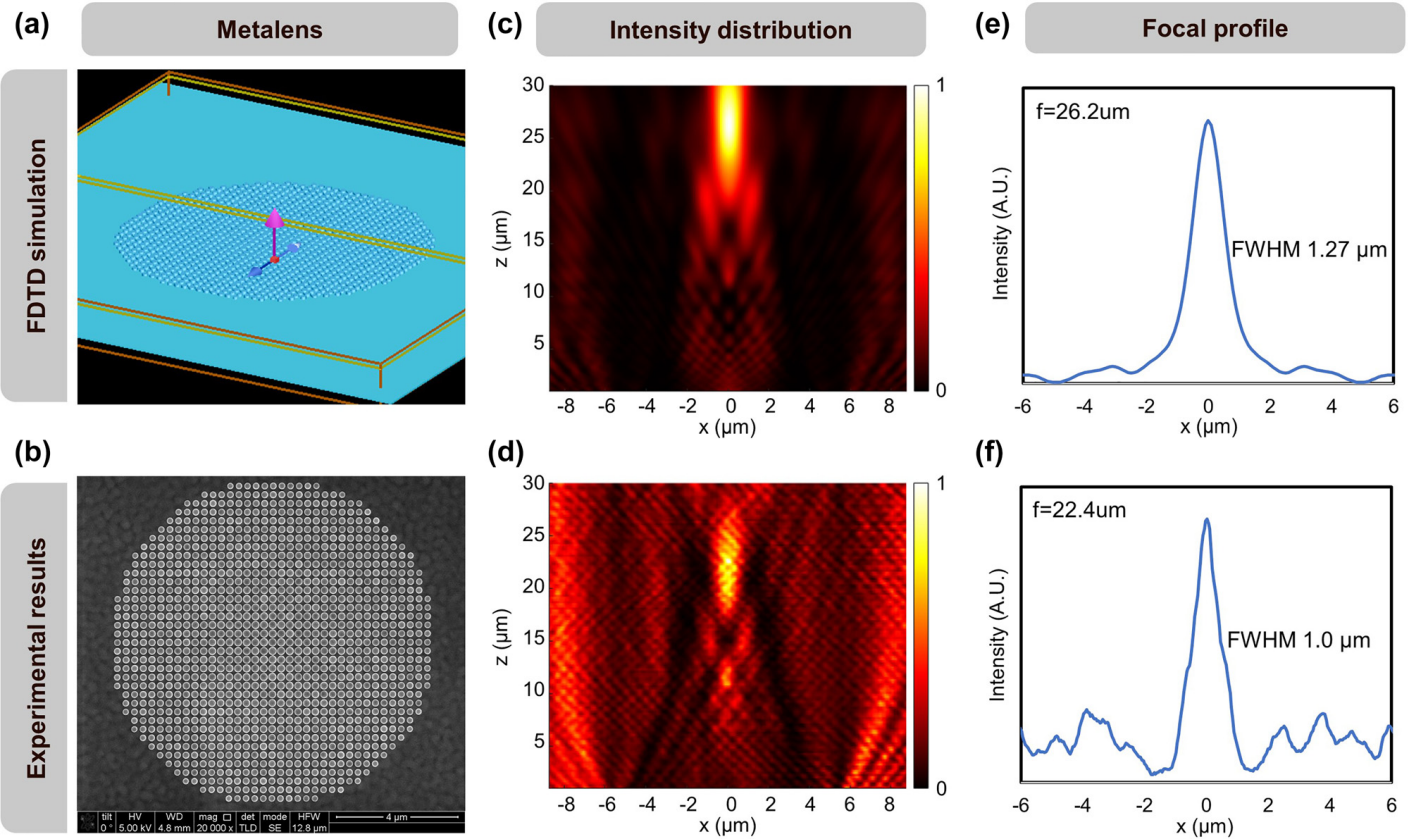
Optical characterization of the metalens operating at 532 nm wavelength. (a) FDTD model of the metalens. (b) SEM micrograph of the fabricated metalens. (c) Simulated electric field intensity distribution of the *x*–*z* plane at *y* = 0. The simulation NA is 0.21. (d) Experimental field intensity distribution of the *x*–*z* plane at *y* = 0. The experimental NA is 0.24. (e) Simulated and (f) experimentally measured focal spot line profile of the metalens.

The monochromatic imaging already demonstrates the lens focusing function, which is further quantified by optical characterization, in comparison with the FDTD model in Figure 5, studying the focal spot of the metalens. FDTD model of the metalens is depicted in Figure 5a showing the TiO_2_ nanodisks selected from the library in Figure 2f, h. SEM micrograph of the fabricated metalens on GeO_2_ (before transfer) is shown in Figure 5b, displaying the nanodisks with variable radius as designed in the FDTD model. To analyze the lens focusing behavior, the electric field intensity distribution was simulated and compared against the imaged field intensity of the fabricated metalens in the *x*–*z* plane as plotted in Figure 5c–d. The simulated focal length is 26.2 μm, smaller than the designed value of 30 μm. The Simulation NA is 0.21, higher than the designed NA of 0.18. This is because the phase profile achieved by the Huygens metasurface is slightly different from the target profile, which can be attributed to the nonlocal interactions between meta-atoms. The experimental intensity distribution is in good agreement with the simulated result, with a smaller focal length of 22.4 μm and a higher NA of 0.24. The deviations can be attributed to material and geometric imperfections in fabrication, which deviate from ideal simulation parameters.

Further insight can be obtained by examining the focal spot. The focal spot line profile of the metalens was both simulated and experimentally measured as depicted in Figure 5e and 5f. The full width at half maximum (FWHM) was calculated based on the focal spot line profile of the metalens. The simulated FWHM is 1.27 μm, close to the diffraction limited value of 1.29 μm at a focal length of 26.2 μm. The experimental FWHM is around 1.0 μm, also close to the diffraction limited value of 1.1 μm at a focal length of 22.4 μm. Overall, the metalens arrays are completely functional for diffraction limited focusing after the transfer process. It highlights that our transfer method is effective in decoupling metasurfaces from substrates while retaining the optical properties on target substrates. Our previous work showed that TiO_2_ Huygens metasurfaces have limited optical efficiency because meta-atom interactions are not considered in conventional intuitive design based on library search. In the future the optical performance of freestanding membrane-based metasurfaces can be improved by using our inverse design strategy [37] or replacing the SiN_*x*_ membrane with a low-refractive-index Al_2_O_3_ membrane [15].

Using Huygens metasurfaces as an example, this study establishes an universal metasurface transfer method. We conclude that our process is compatible with widely used platforms to obtain high quality metasurfaces for inhomogeneous integration. Generally, non-planar substrates can pose extra challenges in ensuring homogenous and conformal contact of the metasurface compared to the case of planar target substrates. Such an effect highlights future challenges and studies. Deterministic transfer with micrometer-scale alignment is promising by further integrating the transfer setup with high resolution micromanipulators for precise alignment and even motorized automation, which will be a key focus for future applications and is beyond the scope of this work.

## 3 Conclusions

In conclusion, we successfully demonstrated a universal PMMA-assisted transfer technique that allows transfer of metasurface nanostructures onto arbitrary substrates with high fidelity. As a proof-of-concept, a Huygens’ metalens operating at a wavelength of 532 nm was initially fabricated on a Si substrate and successfully transferred onto a target substrate, a SiN_*x*_ membrane with 100 nm thickness. Such a transfer enables a metalens with overall thickness (including substrate) of ∼220 nm, which, to the best of our knowledge, is the thinnest in the literature. Optical and SEM characterization have confirmed that the transfer method can well maintain the structural integrity of the metasurface arrays and accomplish the designed optical functionality on a target substrate. By decoupling the fabrication substrate and target substrate, the transfer technique presented here largely extends the conventional metasurface substrates from bulky and rigid planar surfaces to non-planar, flexible, and soft surfaces, and thereby expands their design space and application scenarios. It also paves the way towards cascaded multilayer integration of metasurfaces and their heterogenous integration with various other electronic and photonic devices, such as optical fibers, modulators, sensors, and integrated circuits.

## 4 Methods

### 4.1 Metasurface fabrication and transfer

Initially, a 400-nm thick Ge film was deposited on a Si substrate by electron beam evaporation. The Ge film was then oxidized into GeO_2_ as the sacrificial layer in a tube furnace for 12 h at 550 °C in a dry oxygen flow (12 bubbles/s). The GeO_2_ serves as a water-soluble sacrificial layer and avoids the use of harsh chemical etchants that may damage the metasurfaces during the transfer process. A layer of 120 nm thick TiO_2_ was deposited on the GeO_2_/Si substrate by atomic layer deposition (ALD). A bilayer of e-beam resist PMMA was spin-coated and then sputtered with 10 nm Au as a conductive layer. The coated samples were exposed in an e-beam lithography system (JEOL8100FS). After e-beam exposure, the Au conductive layer was removed by wet etching with Au etchant (standard potassium monoiodide). The samples were developed in (methyl isobutyl ketone) MIBK/isopropanol (IPA) (1:3) at 4 °C with ultrasonication for 1 min. The developed samples were deposited with 10 nm Cr by e-beam evaporation. Cr hard masks for pattern transfer were formed after lift-off. The samples were then processed by TiO_2_ dry etching (SF_6_ gas, 12 mTorr, RF power = 165W, ICP power = 135W). The Cr masks were removed by wet etching. In this way, metasurfaces were formed on fabrication substrates (Si).

To start the transfer process, PMMA was spin-coated on top of the metasurface/GeO_2_/Si substrate at 4000 rpm for 1 min and baked at 80 °C for 5 min. This step was repeated twice. Kapton tapes were then mounted on the edges of the sample, serving as a frame to further minimize deformation and wrinkles of the flexible PMMA film and to facilitate manipulation during the transfer process. To release the metasurface/PMMA assembly layer from Si substrate, samples were immersed in a deionized (DI) water bath at 70 °C for about 10 h, resulting in the dissolution of the sacrificial GeO_2_ film. During the dissolving of GeO_2_ layer, the metasurface/PMMA assembly layer was gradually delaminated from the Si substrate, eventually floating on top of the wafer surface. Subsequently, the metasurface/PMMA assembly layer was transferred onto a target substrate. Here we used a suspended SiN_*x*_ membrane as an example. The suspended SiN_*x*_ membrane was a TEM grid purchased from SPI Supplies, Inc. The SiN_*x*_ membrane was 100 nm thick and grown on a Si wafer with an etched window of ∼100 μm × 100 μm. During this step, the target substrate was mounted at the bottom of the water container (used to dissolve GeO_2_). The water was drained using a syringe and the water surface was continuously lowered. As the water surface is close to the target substrate surface, the orientation and position of the metasurface arrays were manipulated under a microscope using the kapton frame as handles to aid in the alignment process. After the transfer, the metasurface/PMMA layer was baked at 80 °C for about 30 min to improve its adhesion to the target substrate. The PMMA layer was then dissolved in acetone, completing the transfer process.

### 4.2 Optical characterization

The transferred metasurfaces on SiN_*x*_ membranes were imaged in an inverted microscope (Olympus IX73) by white light from a Halogen lamp and a green laser (wavelength 532 nm, Opto Engine) respectively (Figure 4a). A home-built optical set-up was used to introduce the laser from the top of the inverted microscope. Image stacks were taken while moving the stage in the *z*-direction automatically (Prior ES10ZE Focus Controller) and then processed to obtain the *x*–*z* intensity distribution (Figure 5d) and focal spot line profile (Figure 5f).

### 4.3 Numerical simulation

FDTD simulations were carried out using a commercial software (Lumerical). To build a meta-atom database, a single TiO_2_ nanodisk was simulated with periodic boundary conditions in both the *x* and *y* in-plane directions, and varying geometric parameters of disc radius and gap between discs. The incident wave polarization was in the *y* axis. Both transmittance and phase shift were recorded for nanodisks on bulk glass (Figure 2a and b) and SiN_*x*_ membrane (Figure 2d and e), respectively. The transmittance was defined as the transmitted power normalized by the source power. For device simulation, a whole metalens was modeled on a SiN_*x*_ membrane (Figure 5a). The lens *x*–*z* intensity distribution was recorded (Figure 5c), which also provided a focal spot line profile (Figure 5e).
